# Evaluation of perception threshold and pain in patients with Parkinson’s disease using PainVision^®^

**DOI:** 10.3389/fneur.2023.1130986

**Published:** 2023-05-05

**Authors:** Kanako Kurihara, Shinsuke Fujioka, Takayasu Mishima, Yoshio Tsuboi

**Affiliations:** Department of Neurology, Fukuoka University, Fukuoka, Japan

**Keywords:** Parkinson’s disease, pain, PainVision^®^, perception threshold, pain intensity

## Abstract

**Introduction:**

Pain is one of the most frequent non-motor symptoms occurring in patients with Parkinson’s disease (PD). Traditionally, the Visual Analog Pain Scale (VAS), Numerical Rating Scale (NRS), and Wong-Baker Faces Pain Rating Scale (FRS) have been used for clinical pain assessment, but these assessments are subjective at best. In contrast, PainVision^®^ is a perceptual/pain analyzer that can quantitatively evaluate pain as “pain intensity” based on “current perception threshold” and “pain equivalent current.” We evaluated the current perception threshold in all PD patients and pain intensity in PD patients with pain using PainVision^®^.

**Methods:**

We recruited 48 patients with PD (PwPD) with pain and 52 PwPD without pain. For patients with pain, we measured current perception threshold, pain equivalent current, and pain intensity using PainVision^®^, in addition to evaluation by VAS, NRS, and FRS. For patients without pain, only current perception threshold was measured.

**Results:**

There was no correlation with either VAS or FRS, whereas only weak correlation was identified for NRS (*γ* = −0.376) with pain intensity. Current perception threshold was positively correlated with duration of the disease (*γ* = 0.347) and the Hoehn and Yahr stage (*γ* = 0.259). As a quantitative evaluation of pain, pain intensity by PainVision^®^ does not correlate with conventional subjective pain assessments.

**Discussion:**

This new quantitative evaluation method of pain may be suitable as an evaluation tool for future intervention research. Current perception threshold in PwPD was related to the duration and severity of the disease and may be involved in peripheral neuropathy associated with PD.

## 1. Introduction

Pain is one of the most salient non-motor symptoms that afflicts patients with Parkinson’s disease (PwPD), and its frequency varies from 40 to 85%, depending on the report (1–3). Pain can occur at any stage of the disease, from early PD to advanced stages, and some pain is known to precede motor symptoms (4). As the disease progresses, the frequency of pain complications increases due to a variety of factors, including pain associated with motor fluctuation, dyskinesia, dystonia, and postural abnormalities. As it is a subjective sensation, pain has traditionally been considered difficult to quantify. The Visual Analog Pain Scale (VAS) is the most used, conventional pain assessment tool (5) because of its simplicity and ease of use. However, concern has been raised for its use as it is a subjective assessment tool that relies on patient reporting, and these results can easily be swayed by personal experience and psychological factors. In response to this challenge, Shimazu et al. (6) developed an objective method of pain evaluation called PainVision^®^, which is a perceptual/pain analyzer that quantitatively evaluates pain as “pain intensity” based on “current perception threshold” and “pain equivalent current.” PainVision^®^ has contributed to a more objective evaluation of pain regardless of the disease; however, to our knowledge, there are no reports of pain in PD that have been assessed using PainVision^®^. Thus, we closely examined pain in PwPD by quantifying PD pain with PainVision^®^ in addition to using conventional tools of pain assessment.

## 2. Materials and methods

This study was conducted as a single-center, cross-sectional study. We assessed 111 sequential PwPD (57 patients with pain and 54 patients without pain), who received treatment at the Department of Neurology, Fukuoka University Hospital from October 2020 to March 2022. Patients with a definite cause of pain other than PD, such as pain due to arthritis or malignancy, were excluded. All patients were examined by a movement disorder specialist and clinically diagnosed with established PD or probable PD according to the International Parkinson and Movement Disorder Society (MDS) diagnostic criteria (7). Eligible patients were over 20 years old, who understood the purpose and methods of the study, and gave written consent. Exclusion criteria were as follows: (1) patients who could not give consent; (2) patients with severe dementia or psychiatric symptoms that could interfere with the assessment; and (3) patients with electronic devices such as pacemakers or implantable cardioverter defibrillators in their bodies. This study was approved by the Ethical Review Board of Fukuoka University (U20-08-009). Demographic and background information such as age, sex, age at disease onset, duration of disease, wearing off phenomenon, dyskinesia, and hallucinations were extracted from the patient medical records. Levodopa-equivalent daily dose (LEDD) was calculated from the medications according to the standard assessments (8). Motor symptoms were evaluated by a movement disorder specialist using the Hoehn and Yahr (HY) stage (9) and the Movement Disorder Society Unified Parkinson’s Disease Rating Scale (MDS-UPDRS) part III (10). Cognitive function was assessed with the Japanese version of the Montreal Cognitive Assessment (MoCA) (11, 12) and the Mini Mental State Examination (MMSE). The permission of using MoCA was obtained. Depression was assessed using Zung’s Self-Rating Depression Scale (SDS) (13). The 9-symptom Wearing-off Questionnaire (WOQ-9) (14, 15) was used to evaluate the phenomenon of wearing off. In this study, patients were considered to have “wearing off” if they had two or more symptoms positive on the WOQ-9 item and if they improved with dopaminergic therapy. Patients’ quality of life was assessed using the PDQ-8 (16), and their total score (PDQ-8 SUM) and summary index (PDQ-8 SI) were calculated (17). Patients’ clinical subtypes were classified into tremor dominant (TD) subtype, postural instability/gait difficulty (PIGD) subtype, and indeterminate type based on TD scores and PIGD scores of the MDS-UPDRS (18). Pain in PD was qualitatively assessed using the King’s Parkinson’s Disease Pain Scale (KPPS) (19). The types of pain were classified as follows: 1, Musculoskeletal pain; 2, Chronic pain; 3, Fluctuation-related pain; 4, Nocturnal pain; 5, Oro-facial pain; 6, Discoloration, edema/swelling; and 7, Radicular pain (19). Pain was assessed using the VAS (5), Numerical Rating Scale (NRS) (20), and Wong-Baker Faces Pain Rating Scale (FRS) (21). In addition, we assessed current perception threshold, pain equivalent current, and pain intensity using PainVision^®^. For patients without pain, only current perception thresholds were assessed. We performed these evaluations during a patient’s on state.

### 2.1. Scales

- KPPS: This is a scale for evaluating pain specific to PD patients. The KPPS classifies pain into seven domains. In response to the 14 questions, an evaluator will quantify and describe the severity and frequency of the symptom. The score for each item is obtained by multiplying the severity (0–3) by the frequency (0–4). The maximum score is 144, with higher scores indicating more pain (19).
- VAS: This is a scale for evaluating pain numerically. Participants can indicate the degree of pain by marking on a 100 mm line segment ranging from 0 mm of “no pain” to 100 mm of “greatest pain imaginable” (5).
- NRS: This is a scale for evaluating pain numerically. This scale is a 11, 21, or 101 point scale where the end points are the extremes of no pain and the worst pain. Participants point to their current level of pain in numerical terms. The NRS can be graphically or verbally delivered (20). In this study, the NRS was graphically delivered ranging from 0 of “no pain” to 10 of “greatest pain imaginable.”
- FRS: This is a scale for evaluating pain according to a person’s facial expressions. The illustrations of faces are lined up ranging from happy face to crying face. Patients are asked to select an illustration of a facial expression that is similar to their own feelings (21). In this study, we used the scale which shows a series of faces ranging from 0 of “no hurt” to 10 of “hurts worst.”

### 2.2. PainVision^®^

**Figure 1:**
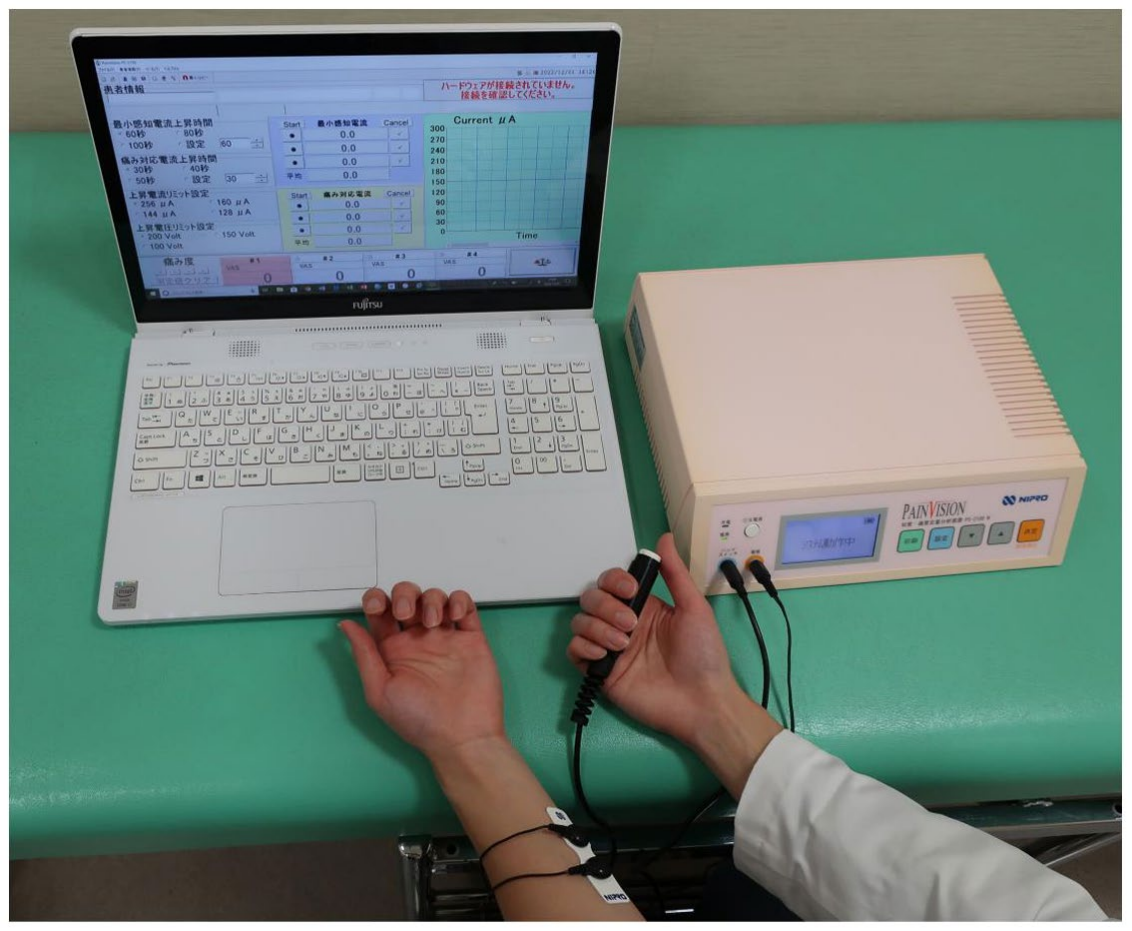
A disposable electrode is attached to the medial forearm, and current is applied to the electrode. The participant presses hand switch to measure “current perception threshold” and “pain equivalent current.”

PainVision^®^ (PS-2100; Nipro Co., Osaka, Japan) is a medical device that can quantify and objectively evaluate degrees of pain. The degree of pain is replaced by a different sensation of current stimulation, which is measured as a current value. This test inflicts low levels of pain because it stimulates a portion of the Aβ and Aδ fibers in the sensory nerves, and less of the C fibers. A disposable electrode EL-BAND is attached to the medial forearm opposite to the dominant hand, and current is applied to the electrode to measure “current perception threshold” and “pain equivalent current” (Figure 1). To measure the current perception threshold, a weak current with a basic cycle of 50 HZ is applied to the electrode and gradually increased. The current perception threshold is measured by pressing a hand switch when the participant feels some stimulus at the electrode. The pain equivalent current is measured by further increasing the current stimulation and the pressing of a hand switch when the participant feels that the degree of pain and the electrode stimulation are equal or greater than the current stimulation. The current perception threshold and the pain equivalent current are each measured three times, and the average value is extracted. Although there were no rules regarding measurement error, the current perception threshold was defined as a value that fell within ±1% of the mean value and the pain equivalent current as a value that fell within ±20% of the mean value in the three measurements. The mean values of the current perception threshold and the pain equivalent current were used to measure the pain intensity = (pain equivalent current − current perception threshold) × 100/current perception threshold (6).

### 2.3. Statistics

Age, age at onset, duration of disease, LEDD, HY stage, UPDRS part III, SDS, MMSE, MoCA, PDQ8-SUM, PDQ-8-SI, and current perception threshold were analyzed by Mann–Whitney U test between PwPD with pain and without pain. Sex, subtype (TD, PIGD, Indeterminate type), wearing off phenomenon, dyskinesia, and hallucinations between the two groups were analyzed by chi-square test. Correlation coefficients between pain intensity and duration of disease, VAS, NRS, and FRS were analyzed using Pearson’s correlation coefficient. The correlations between current perception threshold and age of onset, duration of disease, LEDD, HY stage, and UPDRS Part III were analyzed using partial correlation coefficients after controlling for age. All value of *p*s < 0.05 were considered statistically significant. Data were analyzed by SPSS v.26 (SPSS Inc., Chicago, IL, United States).

## 3. Results

**Figure 2:**
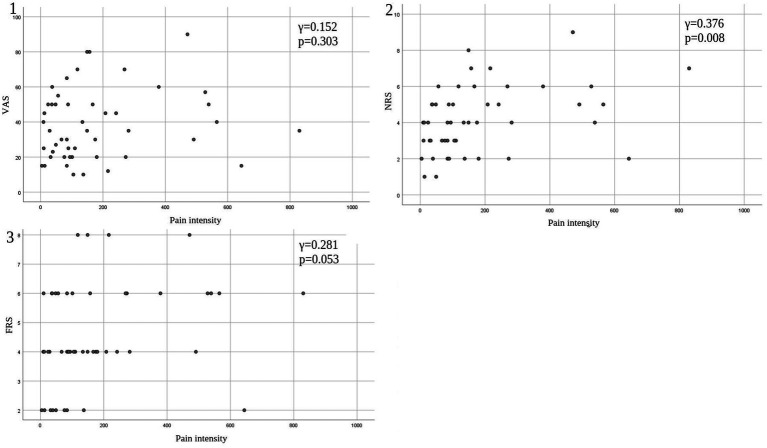
Correlation between pain intensity and conventional tools of pain assessment. (1) Pain intensity versus VAS. (2) Pain intensity versus NRS. (3) Pain intensity versus FRS. VAS, Visual Analog Scale; NRS, Numerical Rating Scale; FRS, Wong-Baker Faces Pain Rating Scale.

**Figure 3:**
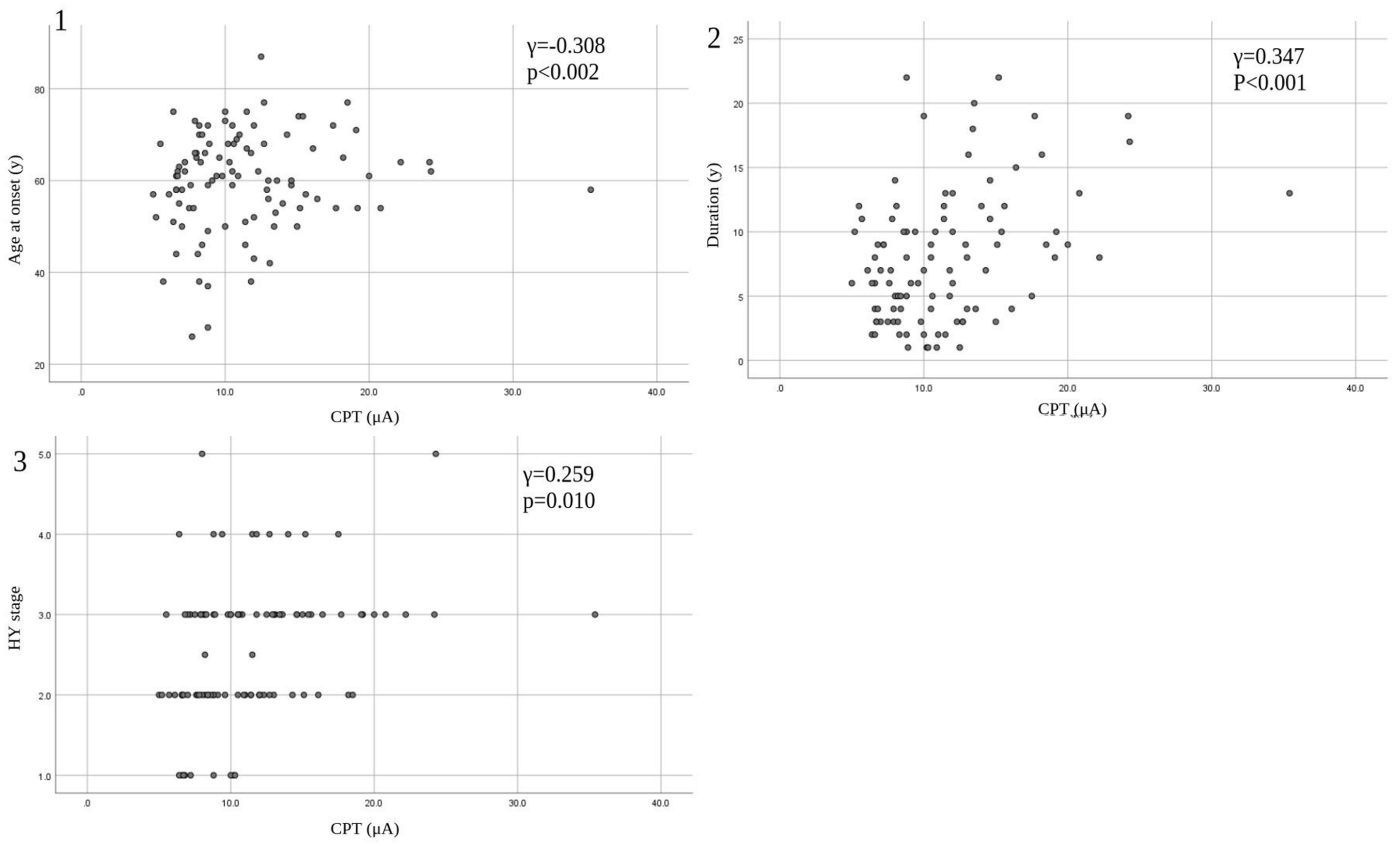
Correlation between CPT and patients’ characteristics. (1) CPT versus age at onset. (2) CPT versus duration. (3) CPT versus HY stage. CPT, current perception threshold; HY, Hoehn and Yahr.

Eleven patients (nine with pain and two without pain) were excluded because seven of these patients had large measurement errors in current perception thresholds or pain equivalent current as measured by PainVision^®^, and four other patients had a definite cause of pain other than PD. Table 1 shows the clinical characteristics of the patients and comparison between PwPD with pain and without pain. The participants were 48 patients with pain and 52 patients without pain. There were 45 males and 55 females, mean age 68.0 ± 10.53 y, mean disease duration 7.95 ± 5.12 y, mean HY stage 2.57 ± 0.85, and mean UPDRS Part III 25.53 ± 12.36. Compared to PwPD without pain, PwPD with pain showed significantly longer disease duration (*p* = 0.038), higher LEDD (*p* = 0.001), higher HY stage (*p* = 0.015), more PIGD subtypes (*p* = 0.004), and higher dyskinesia complications (*p* = 0.045). Table 2 shows details of the background of patients’ with pain. The mean duration of pain was 7.46 ± 8.91 y, and the use of analgesics was 39.6%. The majority of patients (75%) had musculoskeletal pain, and 37.5% had two or more types of pain. Table 3 shows correlation analysis of pain intensity and other factors. Correlation between VAS and FRS was non-significant and weak correlation was identified for NRS (*γ* = −0.376) with “pain intensity” evaluated by PainVision^®^ (Figure 2). Table 4 shows correlation analysis between VAS and other factors. Strong positive correlations were found between VAS and NRS (*γ* = 0.758) and FRS (*γ* = 0.658). Table 5 shows partial correlation coefficient between current perception threshold and other variables after controlling for age. There was a weak negative correlation between current perception threshold and age at onset (*γ* = −0.308), and weak positive correlations with duration of disease (*γ* = 0.347) and HY stage (*γ* = −0.259; Figure 3). No correlation was found between current perception threshold and pain intensity.

**Table 1:** Baseline clinical characteristics of Parkinson’s disease patients with pain and those without pain.

	Total (*n* = 100)	No pain (*n* = 52)	Pain (*n* = 48)	Value of *p*
Sex, male (*n*)	45 (45%)	26 (50%)	19 (39.6%)	0.296
Age (years)	68.0 ± 10.53	68.85 ± 10.71	67.08 ± 10.35	0.472
Age at onset (years)	59.93 ± 10.86	61.79 ± 11.28	57.92 ± 10.12	0.74
Duration (years)	7.95 ± 5.12	6.87 ± 4.44	9.13 ± 5.57	0.038
LEDD (mg)	567.7 ± 354.57	454.29 ± 273.99	690.57 ± 392.06	0.001
HY stage	2.57 ± 0.85	2.37 ± 0.81	2.79 ± 0.85	0.015
UPDRS part III	25.53 ± 12.36	25.35 ± 14.35	25.73 ± 9.92	0.567
TD subtype	37 (37%)	28 (53.8%)	9 (18.8%)	<0.001
PIGD subtype	56 (56%)	22 (42.3%)	34 (70.8%)	0.004
Indeterminate type	6 (6%)	2 (3.8%)	4 (8.3%)	0.301
SDS	42.81 ± 9.25	41.62 ± 8.58	44.1 ± 9.85	0.198
MMSE	28.2 ± 3.36	28.37 ± 4.07	28.02 ± 2.39	0.613
MoCA	24.1 ± 4.69	24.08 ± 4.62	24.15 ± 4.8	0.895
Wearing off (*n*)	47 (47%)	22 (42.3%)	25 (52.1%)	0.328
Dyskinesia (*n*)	30 (30%)	11 (21.2%)	19 (39.6%)	0.045
Hallucination (*n*)	24 (24%)	11 (21.2%)	13 (27.1%)	0.488
PDQ-8 SI	19.62 ± 15.23	17.36 ± 13.41	22.07 ± 16.79	0.162
PDQ-8 SUM	6.28 ± 4.87	5.56 ± 4.29	7.06 ± 5.37	0.175
CPT	11.39 ± 4.92	10.41 ± 3.67	12.44 ± 5.84	0.092

All data are presented as mean ± standard deviation or *n* (%). LEDD, Levodopa equivalent daily dose; HY, Hoehn and Yahr; UPDRS, Unified Parkinson’s Disease Rating Scale; TD, tremor dominant; PIGD, postural instability/gait difficulty; SDS, Self-Rating Depression Scale; MMSE, Mini-Mental State Examination; MoCA, The Montreal Cognitive Assessment; PDQ-8 SI, The Parkinson’s Disease Questionnaire-8 Summary Index; PDQ-8 SUM, The Parkinson’s Disease Questionnaire-8 Sum Score; CPT, current perception threshold.

**Table 2:** Pain evaluations in Parkinson’s disease patients with pain (*n* = 48).

Duration of pain (years)	7.46 ± 8.91
Use of analgesics	19 (39.6%)
**Pain assessment by various scales**	
VAS (mm)	38.31 ± 20.17
NRS	4.19 ± 1.83
FRS	4.58 ± 1.75
**Pain assessment by Pain Vision**^**®**^	
CPT (uA)	12.44 ± 5.84
PEC (uA)	32.76 ± 24.16
Pain intensity	180.9 ± 191.18
**KPPS domains**	
Musculoskeletal pain	36 (75%)
Chronic pain	10 (20.8%)
Fluctuation-related pain	13 (27.1%)
Nocturnal pain	2 (4.2%)
Oro-facial pain	0 (0%)
Discoloration, edema/swelling	3 (6.3%)
Radicular pain	6 (12.5%)
Patients with more than one type of pain	18 (37.5%)

All data are presented as mean (standard deviation) or *n* (%). VAS, Visual Analog Scale; NRS, Numerical Rating Scale; FRS, Wong-Baker Faces Pain Rating Scale; CPT, current perception threshold; PEC, pain equivalent current.

**Table 3:** Pearson product–moment correlation coefficient between pain intensity and other variables.

Pain intensity vs.	Duration	HY stage	UPDRS Part III	SDS	VAS	NRS	FRS
γ	−0.196	−0.277	−0.177	−0.136	0.152	0.376	0.281
Value of *p*	0.182	0.057	0.228	0.358	0.303	0.008	0.053

HY, Hoehn and Yahr; UPDRS, Unified Parkinson’s Disease Rating Scale; SDS, Self-Rating Depression Scale; VAS, Visual Analog Scale; NRS, Numerical Rating Scale; FRS, Wong-Baker Faces Pain Rating Scale.

**Table 4:** Pearson product–moment correlation coefficient between visual analog scale and other variables.

VAS vs.	Duration	HY stage	UPDRS Part III	SDS	NRS	FRS
γ	0.225	0.245	0.225	−0.078	0.758	0.658
Value of *p*	0.125	0.093	0.124	0.599	<0.001	<0.001

HY, Hoehn and Yahr; UPDRS, Unified Parkinson’s Disease Rating Scale; SDS, Self-Rating Depression Scale; VAS, Visual Analog Scale; NRS, Numerical Rating Scale; FRS, Wong-Baker Faces Pain Rating Scale.

**Table 5:** Partial correlation coefficient between current perception threshold and other variables after controlling for age.

CPT vs.	Age at onset	Duration	LEDD	HY stage	UPDRS Part III	Pain intensity
γ	−0.308	0.347	0.171	0.259	0.152	−0.135
Value of p	<0.002	<0.001	0.090	0.010	0.133	0.367

CPT, current perception threshold; LEDD, levodopa equivalent daily dose; HY, Hoehn and Yahr; UPDRS, Unified Parkinson’s Disease Rating Scale.

## 4. Discussion

Taking a different approach from traditional pain assessment tools, PainVision^®^ provides a quantitative assessment of sensory thresholds and existing pain regardless of disease. It quantifies pain as “pain intensity” based on “current perception threshold” and “pain equivalent current.” Objective pain assessment became possible by quantifying the degree of pain as “pain intensity.” In this study, we measured “pain intensity” by using PainVision^®^ in PwPD with pain and found no significant correlation with conventional subjective assessments of pain such as VAS and FRS; furthermore, there was only a weak positive correlation (*γ* = 0.376) with NRS.

Because the VAS is a subjective assessment based on past personal experience of pain, it varies widely among individuals (22). By contrast, “pain intensity” by PainVision^®^ is an objective evaluation tool because it is less susceptible to psychological factors (23, 24). Most PwPD experience neuropsychiatric symptoms such as depression, anxiety, sleep disturbances, psychiatric symptoms, and behavioral and cognitive changes (25). In fact, it has been reported that 35% of PD patients have clinically significant depressive symptoms (26). The VAS assessment of pain may fail to capture the accurate level of pain in PwPD, especially when they are psychologically influenced. Although scales of subjective pain assessment such as VAS, are still important for evaluating patients’ pain, objective assessment by PainVision^®^ should also be incorporated at the same time as it can provide important background information that can impact the outcome of therapeutic intervention. Perceived pain is a mixture of subjectivity and objectivity, and patients’ subjective assessments of pain are thought to be affected by their current mental status. However, PainVision^®^ may be more objectively weighted.

Another advantage of PainVision^®^ is its ease of use; PainVision^®^ can be performed in a short period of time, is minimally invasive to the patient, and has a simple examination procedure. Even PwPD, many of whom are elderly, can operate it simply by pressing a hand switch after detecting the current and the current corresponding to the pain. This study was the first to use PainVision^®^ for PD pain assessment, and we experienced no technical difficulties. This device has been used to assess persistent chronic pain, such as low back pain (27), chemotherapy-induced peripheral neuropathy (28), and pain in herpes zoster (24). Furthermore, it has been used in studies of treatment-related pain, such as evaluating the postoperative pain from single-site laparoscopic colectomy (29) and evaluating the effect of plexus brachialis block on postoperative pain after arthroscopic rotator cuff repair (30). Correlations between “pain intensity” by PainVision^®^ and VAS have been reported in evaluations of various types of chronic pain (31), venous chemotherapy-induced vascular pain (32), and chemotherapy-induced peripheral neuropathy (33). On the other hand, contrary to the results of our study, some studies have shown no correlation between “pain intensity” and VAS (24, 34). In a study that assessed low back pain using the VAS, the McGill Pain Questionnaire (MPQ), and PainVision^®^, the values measured by PainVision^®^ showed consistent results even after repeated calculations and good correlation with MPQ, but no correlation with VAS (34). It is interesting to note that the correlation between PainVision^®^ and subjective pain assessment varies based on the disease.

In this study, partial correlation coefficient after controlling for age showed that current perception threshold had a negative correlation with age at onset, and a positive correlation with duration of disease and HY stage. Current perception thresholds in normal participants are higher in men than in women and increase with age (35). Elevated current perception thresholds are suggestive of sensory neuropathy. Sato et al. (36) and Hiramatsu et al. (37) report that current perception thresholds in diabetic patients are higher than in non-diabetic patients and are useful for detecting minor neuropathy without obvious neurological findings. Goda et al. (38) report that the current perception threshold of hemodialysis patients is higher than that of healthy participants. This study also suggests that the presence of minor peripheral neuropathy in PD may be detectable. The cause of peripheral neuropathy in PD is known to be associated with abnormalities in vitamin B12, methylmalonic acid, and fasting homocysteine, so the metabolic effects of long-term exposure to levodopa may cause peripheral neuropathy (39, 40). It is also reported that small fiber density is decreased in PD and that there is α-synuclein deposition in peripheral nerves on skin biopsy (41). PwPD with peripheral neuropathy are associated with suffering from non-motor symptoms such as cognitive decline, axial motor symptoms, and autonomic symptoms (42), suggesting that peripheral neuropathy develops with PD progression.

In this study, we compared PD patients with and without pain. The group with pain had significantly longer disease duration, higher LEDD, higher HY stage, more PIGD subtype, and a higher rate of dyskinesia complications as background factors. Previous studies report an association between pain in PD and duration of disease (43, 44) and that higher HY stage or higher disease severity is associated with pain severity (45, 46), which is consistent with the results of this study. Regarding dyskinesia and pain, a functional magnetic resonance imaging study showed that dyskinetic PwPD experience increased pain sensitivity and centrally sensitized nociceptive pathways (47). It is speculated that altered pain sensitivity may increase the frequency of pain complications in patients with dyskinesia. Regarding PD subtypes and pain, there is a report that pain is associated with the PIGD subtype because it involves more advanced dopaminergic striatal denervation, and dopamine deficiency causes hyperalgesia (48).

The first limitation of this study is that it was a single-center, small-scale study. More patients need to be evaluated with PainVision^®^. Second, the degree of pain was not compared to other assessment methods such as the McGill Pain Questionnaire. Third, though pain in PD is heterogeneous and classified into seven classifications of the KPPS, individual analysis of pain was not performed in this study. Most previous reports of Pain Vision^®^ show that it can measure the degree of nociceptive or neuropathic pain. However, pain in PD has a wide variety of causes, including a lowered pain threshold to nociceptive stimuli and activation of ascending pain pathways (49), and reduced descending pain inhibition (50), which lead to difficult aspects in the interpretation of measurements. Therefore, the type of pain that is most useful for evaluation by PainVision^®^ should be considered in the future.

Despite the above mentioned limitations, we believe PainVision^®^, which enables objective evaluation that is less susceptible to psychological influences, is a useful tool for the evaluation of pain in PwPD. However, as pain in PD is complex, further study is warranted.

## Data availability statement

The original contributions presented in the study are included in the article/supplementary material, further inquiries can be directed to the corresponding author/s.

## Ethics statement

The studies involving human participants were reviewed and approved by Ethical Review Board of Fukuoka University (U20-08-009). The patients/participants provided their written informed consent to participate in this study.

## Author contributions

KK: methodology, statistical analysis, investigation, and writing – original draft. TM and SF: review and editing. YT: conceptualization, formal analysis, and supervision. All authors contributed to the article and approved the submitted version.

## Funding

Financial disclosures for the previous 12 months: YT received personal fees from Eisai Co., Ltd., Takeda Pharmaceutical Co., Ltd., Novartis Pharma, Sumitomo Dainippon Pharma Co., Ltd., AbbVie GK, Otsuka Pharmaceutical Co., Ltd., and Kyowa Kirin Co., Ltd., outside the submitted work. KK and YT were supported by a grant from Nipro Corporation.

## Conflict of interest

The authors declare that the research was conducted in the absence of any commercial or financial relationships that could be construed as a potential conflict of interest.

## Publisher’s note

All claims expressed in this article are solely those of the authors and do not necessarily represent those of their affiliated organizations, or those of the publisher, the editors and the reviewers. Any product that may be evaluated in this article, or claim that may be made by its manufacturer, is not guaranteed or endorsed by the publisher.
